# Integrated transcriptome and DNA methylome analysis reveal the biological base of increased resistance to gray leaf spot and growth inhibition in interspecific grafted tomato scions

**DOI:** 10.1186/s12870-024-04764-8

**Published:** 2024-02-21

**Authors:** Ce Liu, Yanhong Jia, Lixia He, Hui Li, Jian Song, Lizhu Ji, Chunguo Wang

**Affiliations:** 1 College of Life Sciences, Nankai University, Tianjin, 300071 China; 2 Tianjin Academy of Agricultural Sciences, Tianjin, 300380 China; 3 College of Horticulture and Landscape, Tianjin Agricultural University, Tianjin, 300384 China

**Keywords:** Tomato, Grafting, Gray leaf spot, Growth-defense tradeoff

## Abstract

**Background:**

Grafting is widely used as an important agronomic approach to deal with environmental stresses. However, the molecular mechanism of grafted tomato scions in response to biotic stress and growth regulation has yet to be fully understood.

**Results:**

This study investigated the resistance and growth performance of tomato scions grafted onto various rootstocks. A scion from a gray leaf spot-susceptible tomato cultivar was grafted onto tomato, eggplant, and pepper rootstocks, creating three grafting combinations: one self-grafting of tomato/tomato (TT), and two interspecific graftings, namely tomato/eggplant (TE) and tomato/pepper (TP). The study utilized transcriptome and DNA methylome analyses to explore the regulatory mechanisms behind the resistance and growth traits in the interspecific graftings. Results indicated that interspecific grafting significantly enhanced resistance to gray leaf spot and improved fruit quality, though fruit yield was decreased compared to self-grafting. Transcriptome analysis demonstrated that, compared to self-grafting, interspecific graftings triggered stronger wounding response and endogenous immune pathways, while restricting genes related to cell cycle pathways, especially in the TP grafting. Methylome data revealed that the TP grafting had more hypermethylated regions at CHG (H = A, C, or T) and CHH sites than the TT grafting. Furthermore, the TP grafting exhibited increased methylation levels in cell cycle related genes, such as DNA primase and ligase, while several genes related to defense kinases showed decreased methylation levels. Notably, several kinase transcripts were also confirmed among the rootstock-specific mobile transcripts.

**Conclusions:**

The study concludes that interspecific grafting alters gene methylation patterns, thereby activating defense responses and inhibiting the cell cycle in tomato scions. This mechanism is crucial in enhancing resistance to gray leaf spot and reducing growth in grafted tomato scions. These findings offer new insights into the genetic and epigenetic contributions to agronomic trait improvements through interspecific grafting.

**Supplementary Information:**

The online version contains supplementary material available at 10.1186/s12870-024-04764-8.

## Background

Tomato (*Solanum lycopersicum* L.) is a widely cultivated crop with the global production reaching 189.1 million tons in 2021 (FAOSTAT, 2021). The extensive cultivation of tomatoes has led to the continuous apprehensions regarding the diseases and environmental risks in tomato production. The gray leaf spot is one of the major diseases which profoundly reduce tomato yield and quality. The grey leaf spot disease is not detectable in its early infection; therefore, it causes irreversible damage in the later stages. It is of major concern as it is not easily detectable in the early stages and leads to irreversible damage by causing extensive leaf necrosis in the later stages. [1]. The grey leaf spot cause leaf necrosis and is caused by pathogens from the species of *Stemphylium* family and impacts various *Solanaceae* crops [2–4]. Several strategies have been adopted to defend against gray leaf spot such as the selection of resistant cultivars [5]; however, these strategies are either time-consuming or have limited effects in the short term.

Grafting is a traditional agricultural practice that leverages the advantages of rootstocks to improve the resistance performance, making it a potential agronomic approach to enhance tomato resistance against diseases and environmental stresses [6, 7]. Several studies have shown that grafting in vegetables can significantly reduce diseases and environmental risks, especially in successive cropping systems [8]. In *Cucurbitaceae* family, pumpkins are used as rootstocks to enhance cold tolerance in cucumber seedlings by boosting the phenylpropanoid metabolism and phytohormones synthesis [9]. The use of resistant rootstocks in *Rosaceae* family reveals high resistance of apple scions to apple specific replant disease [10]. In *Solanaceae* crops, using *Solanum torvum* as rootstocks can reduce heavy metal stress by lowering the accumulation of cadmium ions in tomato, eggplant, and pepper scions [11]. Drought-tolerant tomato rootstocks can enhance the drought tolerance of drought-sensitive tomato scions by regulating genes involved in ABA biosynthesis and signaling [6], and *Solanum habrochaites* rootstocks can improve the cold tolerance of tomato scions [12]. Grafting in vegetables has been studied extensively in improving plant growth and yield under biotic and abiotic stress. However, the regulatory mechanism of *Solanaceous* grafting on the effect of scion resistance, quality, and yield needs to be further explored.

Previous extensive studies have demonstrated that the rootstock-scion union undergoes multiple processes after grafting events, such as initial adhesion, callus proliferation, rootstock-scion interaction, and vascular reconnection. The wounding process is induced in the callus proliferation and scion-stock interaction stages, causing the upregulation of resistance-related genes like *PTI5* during this phase [13]. Among these processes, $\beta$-1,4-glucanases play an important role in the rootstock-scion adhesion, which is essential for successful grafting [14]. Besides, rootstock-scion interactions can trigger plant endogenous immunity by activating pattern-triggered immunity (PTI) and effector-triggered immunity (ETI), which leads to reactive oxygen species (ROS) bursts [15] and immune-related apoptosis [16]. Furthermore, grafting can induce changes in the epigenetic information of scions, affecting the expression of growth and resistance-related genes indirectly through DNA methylation and acetylation [17, 18]. In fact, rootstock-scion interaction has a lasting effect on the reshaping of the scion's phenotype during *Solanaceous* grafting, although the mechanisms behind it are not fully understood.

To explore the potential of *Solanaceous* rootstocks in conferring resistance to gray leaf spot in tomato grafting, this study used gray leaf spot-susceptible tomato as the scion, tomatoes, eggplants, and peppers as rootstocks for grafting. TT, TE, and TP grafting combinations were created to test the susceptibility of gray leaf spot. Enhanced resistance to gray leaf spot was observed in both TE and TP graftings, though the growth states was severely inhibited in the TP grafting. Phenotypic, physiological, transcriptomic, and DNA methylation analyses were combined to reveal the mechanism of the enhanced resistance and inhibited growth performance observed in the TE and TP graftings compared to the TT grafting. Besides, the consideration of tradeoffs between growth and defense resources in the *Solanaceous* grafting was also discussed.

## Results

### Interspecific graftings using eggplants and peppers as rootstocks increased the resistance of the tomato scion to gray leaf spot

**Fig. 1:**
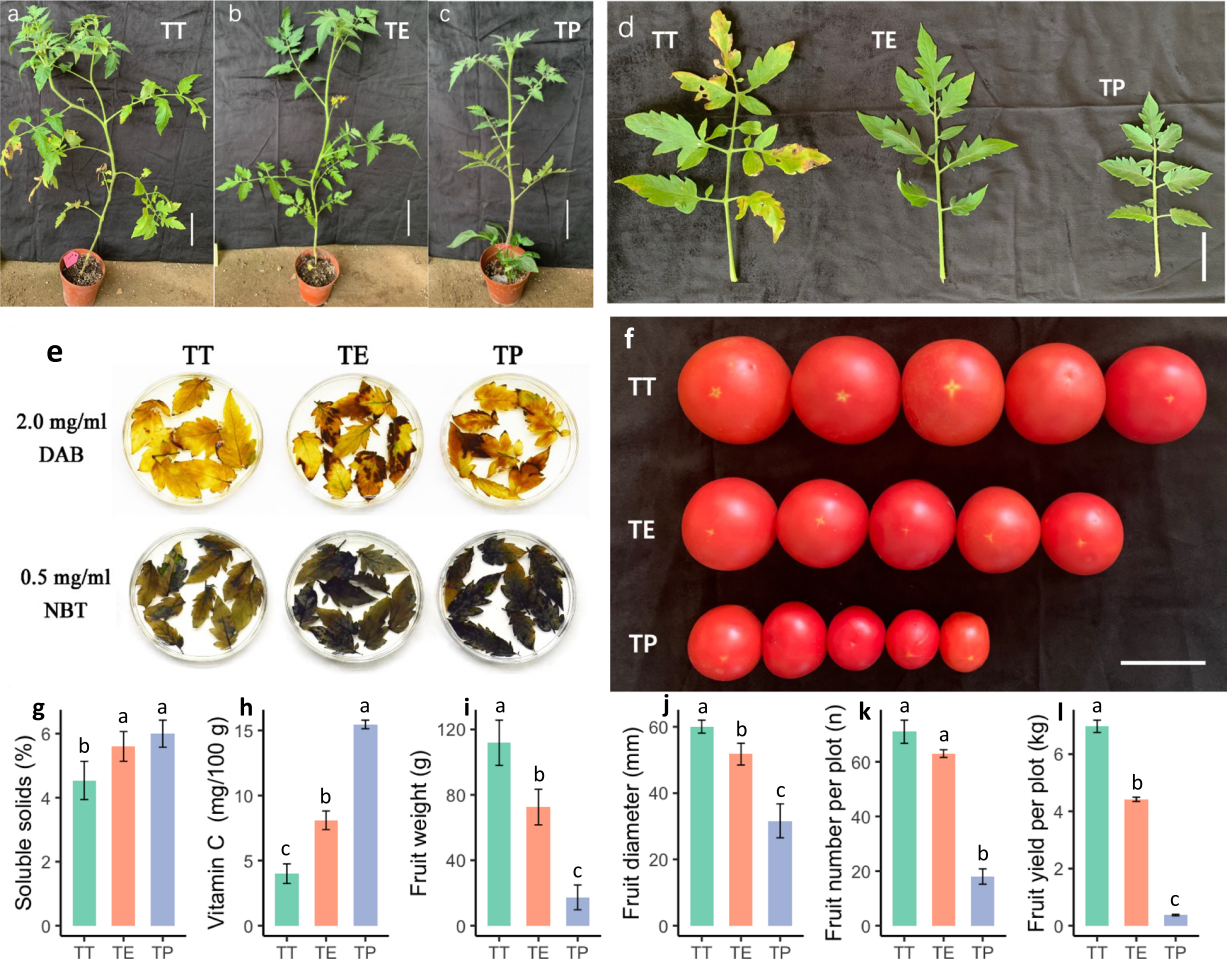
Comparative analysis of pathogen resistance, ROS accumulation, and growth characteristics in the TT, TE, and TP grafted tomato scions. **a**–**d** Disease symptoms in grafted scions 14 days post-inoculation with *Stemphylium* spore suspension. **e** ROS levels, including superoxide anion (O_2_^−^) and hydrogen peroxide (H_2_O_2_), detected in scion leaves using NBT and DAB staining methods, respectively. **f** Mature fruit of tomato scions. **g**–**l** Quantitative analysis of soluble solids and Vitamin C content, single fruit weight, fruit diameter, average number of fruits, and yield per plot in the TT, TE, and TP graftings. Data in g–l are presented as mean ± SD, with different letters denoting statistically significant differences (*p* < 0.05, Duncan's New Multiple Range Test). Scale bars represent 10 cm in a–c and 5 cm in d and f

To evaluate the pathogen resistance of tomato scion grafted on different rootstocks, the scion leaves of TT, TE, and TP grafting combinations were infected by *Stemphylium*, which could result in gray leaf spot. Observations revealed that 14 days post-inoculation, interspecific graftings including TE and TP graftings both displayed resistance to gray leaf spot, manifesting no lesions. While the TT grafting presented typical gray leaf spot symptoms, including leaf yellowing and the emergence of grayish-brown lesions with holes (Fig. 1a–d). The results of 3,3'-diaminobenzidine (DAB) and nitroblue tetrazolium (NBT) staining confirmed that the scion leaves of the TE and TP graftings became darker due to the higher levels of ROS (H_2_O_2_ and O_2_^−^) as compared to that of the TT grafting (Fig. 1e).

### Interspecific grafted tomato exhibited increased fruit quality with inhibited growth

The growth characteristics and fruit quality of different tomato scions were evaluated, and the results indicated that the soluble solid content was significantly higher in the fruits of tomato scions grafted onto eggplants and peppers than those of the self-grafted tomato (Fig. 1g). Moreover, the content of vitamin C in the tomato fruits of TE and TP graftings was significantly higher than in the fruits of TT grafting (Fig. 1h). Nevertheless, compared with the self-grafted tomato, the growth of tomato scions grafted on eggplants and pepper were inhibited, which result in smaller fruit size and lower yield (Fig. 1f, i-l). These results confirmed that in addition to improved pathogen resistance to gray leaf spot, interspecific graftings used eggplants and pepper as rootstocks also could significantly increase the fruit quality of tomato scions, although the fruit yield of these scions was decreased compared with that of the self-grafting tomato.

### Gene expression profiling and genes with significantly different expression levels were identified in the TT, TE, and TP grafting combinations

**Fig. 2:**
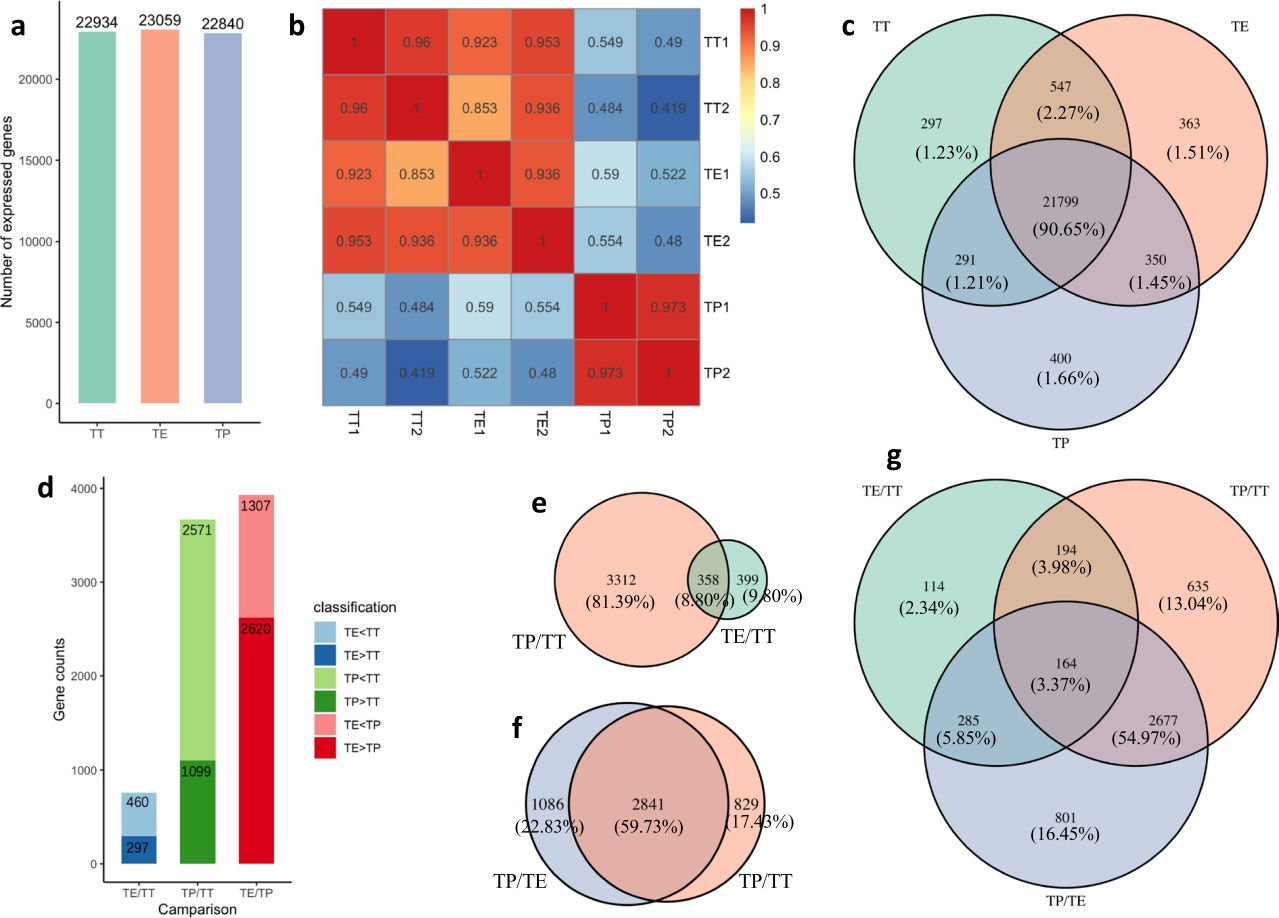
Gene expression profiles and differential expression genes identified in the TT, TE, and TP grafting combinations. **a** Total number of expressed genes in each of the three graftings. **b** Pearson correlation coefficient heatmap depicting the relationship among the samples from the TT, TE, and TP graftings. **c** Venn diagram showing the unique and overlapping expressed genes among the TT, TE, and TP graftings. **d** Bar plot indicating the number of upregulated and downregulated DEGs in each grafting, comparing TT vs TE, TT vs TP, and TE vs TP. **e**–**g** Venn diagrams illustrating the overlap of DEGs in comparisons of TP vs TT, TE vs TT, and TP vs TE, respectively. DEGs were identified based on the criteria of |log_2_ (fold change)|> 2 and q value < 0.001

RNA-seq was performed to uncover the gene expression profiling of tomato scions grafted on tomatoes, eggplants, and peppers, respectively. Sequencing data indicated that a total of 38.78 Gb of high-quality clean bases with a Q30 ratio ranging from 90.76% to 91.56% was obtained (Table S1), and mapped to 22,934, 23,059, and 22,840 genes in the samples of the TT, TE, and TP grafting combinations, respectively (Table S2; Fig. 2a). The sample correlation analysis revealed that the gene transcription levels had a strong correlation within each grafting combination (*r* > 0.93, Fig. 2b), which indicated that the RNA-seq data obtained in the present study were reliable and could be used in the following analysis. Further data analysis indicated that most of the detected genes (21,799 out of 24,047) showed common transcription expression in scions of three grafting combinations. The grafting combination-specific expression genes were obviously rare and only 297, 363, and 400 genes exhibited specific expression patterns in the samples of TT, TE, and TP graftings, respectively (Fig. 2c).

Furthermore, the gene differential expression profiling of tomato scions grafted on three different rootstocks were identified (Table S3; Fig. 2d). Compared with self-grafted tomatoes (TT grafting), 757 genes displayed differential expression levels, of which about 60% (460/757) were downregulated in the scions of the TE grafting. A total of 3670 differentially expressed genes (DEGs) were identified between the scions of the TP and TT graftings. Similarly, a large portion (2571/3670) of the DEGs was downregulated in the scions of the TP grafting compared to those of the TT grafting. The comparative analysis between the TE and TP graftings also indicated that more genes (2620 out of 3927, about 67%) showed downregulated expression trends in the scions of the TP grafting (Fig. 2d). Among these DEGs detected by pairwise comparative analysis in the TT, TE, and TP graftings, 358 overlapping DEGs were confirmed in both TE/TT and TP/TT (Fig. 2e), while 2841 genes showed differential expression levels both in the TP/TE and TP/TT grafting comparisons (Fig. 2f). Only 164 genes showed differential expression levels among three grafting combinations (Fig. 2g). The gene expression profiling and DEGs provided an important clue to further elucidate the gene regulatory network associated with the phenotype of increased tomato scion resistance to gray leaf spot and the inhibition of scion growth in interspecific grafting combinations.

### Gene ontology (GO) and Kyoto Encyclopedia of Genes and Genomes (KEGG) enrichment analysis of the DEGs involved in the interspecific grafting versus self-grafting

The GO and KEGG enrichment analyses were performed to elucidate the potential functions of DEGs in interspecific graftings compared to self-grafting, as presented in Table S4. In the upregulated DEGs of this comparison, a few proteinases inhibitor-related DEGs were significantly annotated in enzyme inhibitor-related GO categories, including "serine-type endopeptidase inhibitor activity", "endopeptidase regulator activity", and "enzyme inhibitor activity" in both TE/TT and TP/TT grafting comparisons. The "plant hormone signal transduction" KEGG pathway also showed substantial enrichment, with 33 DEGs identified in TE/TT and 63 DEGs in TP/TT grafting comparisons. Within these DEGs, 19 DEGs in TE/TT were auxin-responsive proteins, and 7 DEGs in TP/TT were putative late blight resistance proteins. Additionally, 11 DEGs associated with wound-induced proteinase inhibitors were categorized under "response to wounding" GO terms specifically in the TP/TT grafting comparison, as illustrated in Fig. S1a-b. Furthermore, 12 DEGs in TE/TT and 45 DEGs in TP/TT grafting comparisons were annotated in the "MAPK signaling pathway—plant" KEGG pathways, suggesting a potential connection of these pathways with increased immunity to gray leaf spot in interspecific graftings. Remarkably, a subset of upregulated DEGs, isolated based on a |log_2_ (TP/TT)| > 0.8 criterion in the TP/TT grafting comparison, were found to be annotated in GO terms pertinent to jasmonic acid (JA) biosynthesis and metabolic processes, which is provided in Table S5.

Among the downregulated DEGs for the interspecific graftings compared to self-grafting, seven DEGs related to glutathione transferases and twelve DEGs related to cytochrome P450 in the TE/TT grafting comparison were significantly annotated in "detoxification" and "secondary metabolite biosynthetic process" GO terms, respectively. Moreover, KEGG pathways related to secondary metabolites, such as "isoflavonoid biosynthesis", "flavone and flavonol biosynthesis", "phenylpropanoid biosynthesis", and "flavonoid biosynthesis", were significantly enriched in the TE/TT grafting comparison (Fig. S1c). Interestingly, in the TP/TT grafting comparison, 149 GO terms were found to be significantly enriched among the downregulated DEGs. The majority of these GO terms pertained to cell cycle-related processes, such as (1) DNA replication ("DNA replication", "DNA replication initiation", "nuclear replication fork"), (2) cytoplasmic division ("microtubule-based movement", "movement of cell or subcellular component"), and (3) cell wall synthesis ("cell wall organization or biogenesis", "cell wall", "cellulase activity", "lignin catabolic process", "pectin catabolic process"). Furthermore, KEGG pathways related to nutrient metabolisms, such as "fatty acid biosynthesis" and "starch and sucrose metabolism", were also significantly enriched in the TP/TT grafting comparison (Fig. S1d).

### Key genes involved in JA biosynthesis were upregulated in the TP grafting combination

**Fig. 3:**
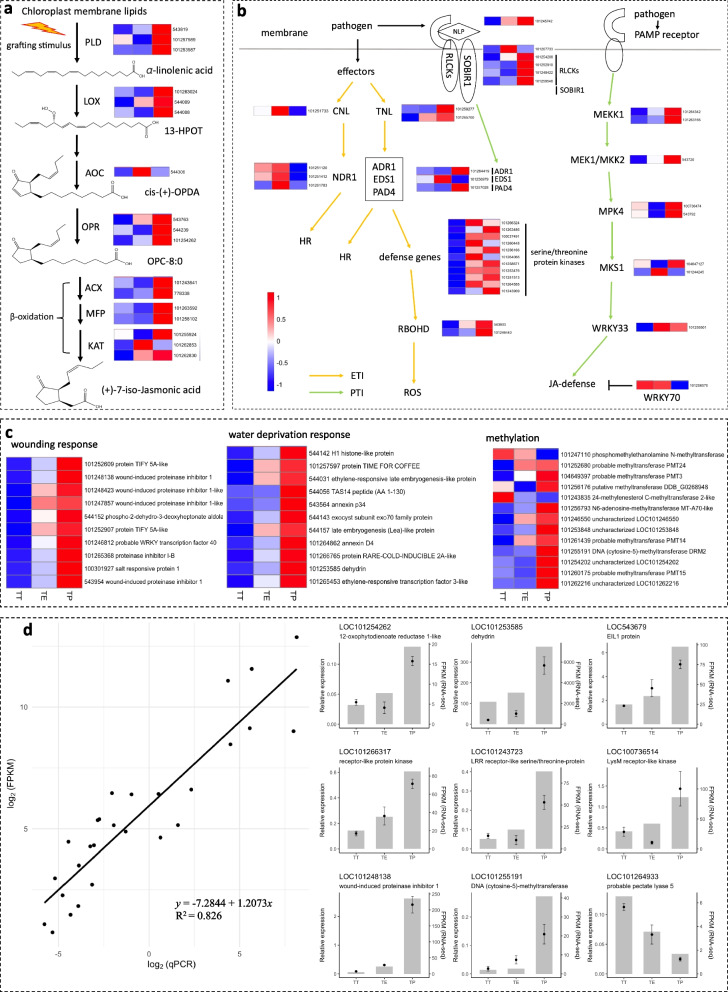
Gene expression analysis of DEGs in pathways related to resistance. This figure highlights the significant shifts in gene expression levels within critical resistance-related pathways in different grafting combinations. **a** DEGs associated with the jasmonic acid synthesis pathway. **b** DEGs pertaining to Effector-Triggered Immunity (ETI) and Pathogen-Triggered Immunity (PTI) pathways. **c** DEGs related to wounding, water dehydration, and methylation responses. **d** qRT-PCR was employed to validate the expression levels of genes associated with defense responses across the TT, TE, and TP graftings. Bar graphs depict the Fragments Per Kilobase of transcript per Million mapped reads (FPKM) values from RNA-seq data, and dots with error bars illustrate the mean and standard deviation values from qRT-PCR results. Heat maps in panels a-c use colors to indicate normalized Z-score values, with specific colors corresponding to specific ranges of expression levels; blocks within the heat maps, from left to right, represent the TT, TE, and TP graftings, respectively. The DEGs depicted in Fig. 3a were selected using a threshold of |log_2_(fold change)|> 0.8 and a q value < 0.001. In other parts of the study, DEGs were identified using a threshold of |log_2_(fold change)|> 2 and a q value < 0.001

Upon examining the transcripts of key genes annotated in the JA biosynthetic pathway, a significant up-regulation was evident in the TP grafting compared to the TT grafting (Fig. 3a). The upregulated genes included the injury-induced phospholipase D (*PLD*), lipoxygenases (*LOX*) performing oxygenation, cyclization, and reduction, allene oxide cyclase (*AOC*), 12-oxophytodienoate reductase (*OPR*), as well as genes involved in β-oxidation, such as acyl-coenzyme A oxidase (*ACX*), multifunctional protein (*MFP*), and ketoacyl-CoA thiolase (*KAT*). This consistent upregulation suggests a sustained heightened JA biosynthesis in the TP grafting post the grafting event.

### A few DEGs involved in immunity and wounding pathways were upregulated in interspecific graftings

Based on the significantly enriched MAPK signaling pathway for the up-regulated DEGs in interspecific graftings versus self-grafting, a further analysis of gene expression in kinase-involved endogenous immune pathways was conducted, which included the DEGs in PTI and ETI. In the PTI pathway, *MEKK1*, *MKK2*, *MPK4*, *MKS1*, and the resistance-related transcription factor *WRKY33* all showed elevated expression in both TE and TP compared to TT grafting (Fig. 3b). Conversely, *WRKY70*, known to suppress JA defense responses, was downregulated in the TP grafting. For the ETI pathway, transcripts of TIR-NBD-LRR (*TNL*) and key signaling components including *EDS1*, *PAD4*, *ADR1*, and several defense-related kinases were predominantly expressed in both TE and TP graftings. However, only TE grafting showed heightened expression of CC-NBD-LRR (*CNL*) and its downstream gene, *NDR1*.

Besides, the upregulated DEGs in the TE/TT and TP/TT grafting comparisons distinctly exhibited a robust genetic response to wounding and dehydration. For instance, JA-induced proteinase inhibitors related to wounding, dehydrins linked to plant dehydration response, and stress-responsive annexins all showed heightened expression in the TE and TP graftings compared to the TT grafting (Fig. 3c). This observation implies an augmented stress on tomato scions in interspecific graftings compared to self-grafting. Notably, the transcript levels of methyltransferase genes, including *PMT13*, *PMT14*, *PMT15*, *PMT24*, and *DRM2*, were more pronounced in the TP grafting than in the TT grafting (Fig. 3c), hinting at potential differences in DNA methylation levels between the graftings. Additionally, the expression of some defense-related genes was validated using quantitative reverse-transcription PCR (qRT-PCR) (Fig. 3d), which confirms the accuracy of gene transcription from RNA-seq.

### A series of genes involved in cell cycle-related processes were severely inhibited in the TP grafting combination

**Fig. 4:**
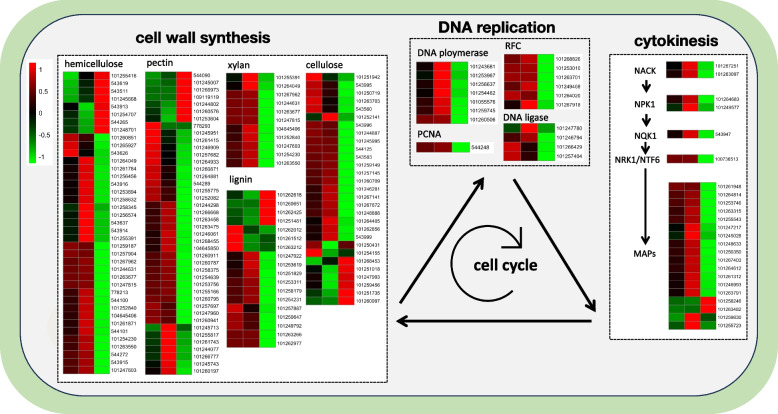
Effects of different grafting combinations on cell cycle processes in tomato scions. TT, TE, and TP graftings were analyzed for their impact on the key genes involved in the cell cycle. The TP grafting significantly inhibited cell cycle processes, including DNA replication, NACK pathway-based cytokinesis, and cell wall synthesis. In the DNA replication component, Replication Factor C (*RFC*) serves as a key player in DNA clamp loading, and Proliferating Cell Nuclear Antigen (*PCNA*) functions as a central component of the replication fork. The heatmap displays normalized Z-score values, with each color corresponding to the Z-score value: the red color indicates higher activity, and the green color represents lower activity. Blocks in the heatmap, from left to right, correspond to the TT, TE, and TP graftings, respectively. The observed inhibition in the TP grafting underscores potential challenges in graft compatibility, highlighting the need for further investigation into compatible graftings

Given the significant enrichment of cell cycle-related GO terms among the downregulated DEGs in the TP/TT grafting comparison, further investigation was conducted into the TP grafting, revealing notable findings. Within the TP grafting, there was a pronounced downregulation of DEGs related to the cell cycle, including those involved in DNA replication, cytokinesis, and cell wall synthesis, as compared to the TT grafting (Fig. 4). For instance, essential components of the DNA replication process, such as genes associated with DNA polymerase, DNA ligase, replication factor C (*RFC*), and proliferating cell nuclear antigen (*PCNA*), were significantly downregulated in the TP grafting. Additionally, genes related to the NACK-PQR pathway, which is critical for cytokinesis initiation, including *NACK*, *NPK*, *NQK1*, *NRK1*/*NTF6*, and *MAPs*, were also significantly downregulated. Furthermore, there was a marked decrease in the genes associated with cell wall biosynthesis, including those involved in the metabolism of hemicellulose, pectin, xylan, lignin, and cellulose, in the TP grafting (Fig. 4).

Moreover, a trend of declining transcription levels was observed in genes encoding ribosomal subunits, such as 30S, 40S, 50S, and 60S ribosomal proteins, across TT, TE, and TP graftings. This decline was also evident in the genes related to chlorophyll proteins involved in photosynthesis within interspecific graftings (Fig. S2). Collectively, these observations underscore a comprehensive downregulation of key cellular processes in the TP grafting, revealing the potential mechanism for inhibited growth and decreased fruit yield observed in interspecific graftings.

### TP grafting displayed a higher number of hypermethylated regions at CHG and CHH sites compared to TT grafting

Complementing the transcriptomic data, an investigation into the DNA methylation variations across the three grafting combinations was conducted, which was critical for unraveling the complex mechanisms contributing to the enhanced resistance against gray leaf spot observed in the interspecific graftings. A total of 150 Gb of high-quality whole-genome bisulfite sequencing (WGBs-seq) bases of TT, TE, and TP grafting combinations were generated after filtering out low-quality reads. Sample mapping rates varied ranged from 81.04% to 85.41%, and bisulfite conversion rates exceeded the 99% in all cases, qualifying the data for further methylation analysis (Table S6). An examination of CG methylation within individual chromosomes across the three grafting combinations revealed a consistent pattern: high CG methylation, moderate CHG methylation, and low CHH methylation (Table S7; Fig. S3a). Notably, chloroplast DNA methylation stood out for its scarcity in all contexts (CG, CHG, and CHH) at extremely low levels (< 3%). A comparative analysis exhibited no obvious differences in CG methylation among the three graftings. However, both CHG and CHH methylation presented a notable uptick in the TP grafting compared to the TT grafting, with the discrepancy particularly pronounced for CHH methylation (Fig. S3a–b).

### Distinct methylation patterns across genomic regions in the TT, TE, and TP grafting combinations

**Fig. 5:**
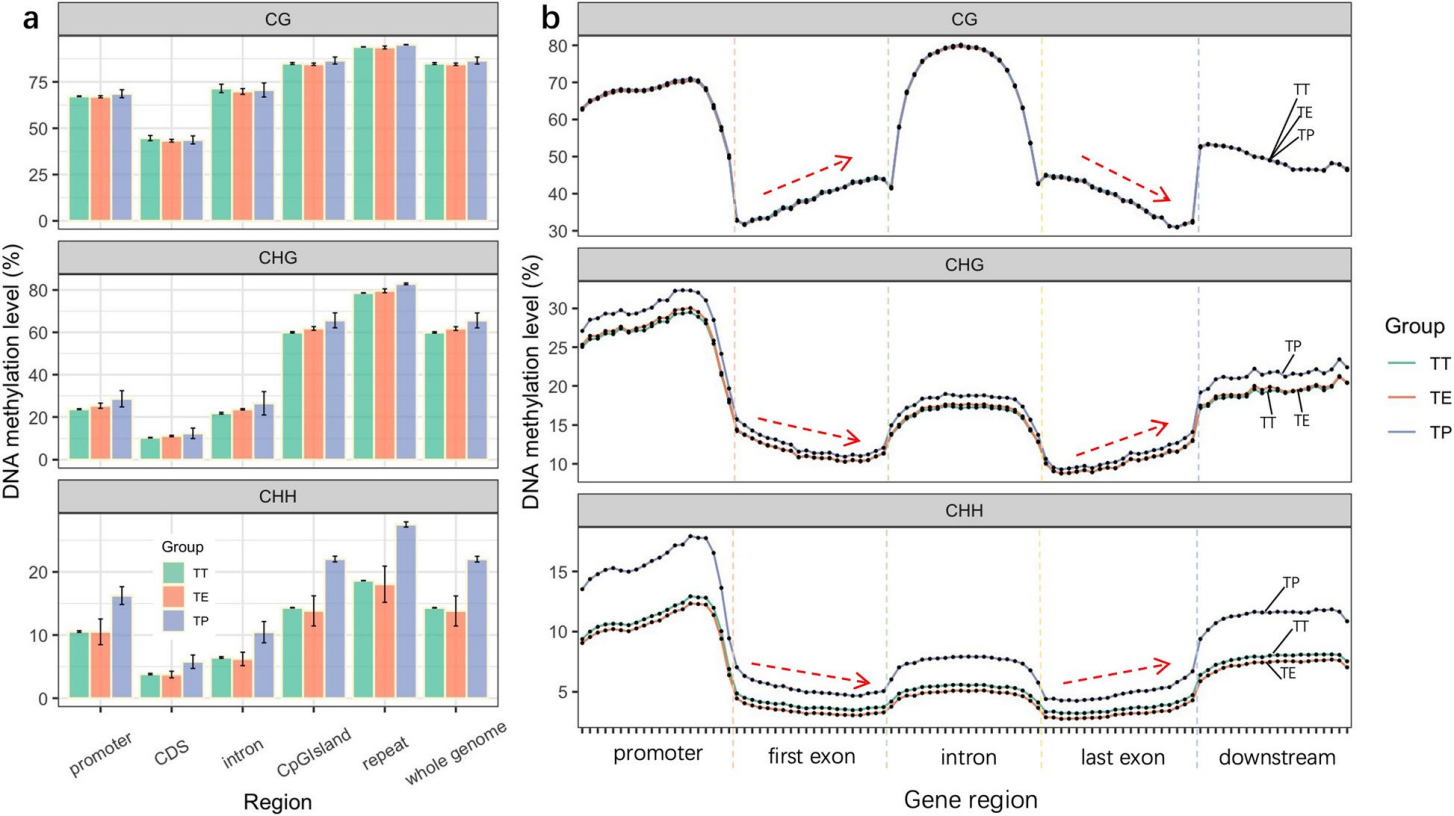
Comparative DNA methylation patterns across various regions in the TT, TE, and TP graftings. **a** The methylation levels in different genomic regions, encompassing the promoter, CDS, intron, CpG island, repeat regions, and the entire genome, highlighting the contrasts among the three grafting types. **b** The average trends in DNA methylation across different functional gene regions, including the promoter, first exon, intron, last exon, and downstream regions. For analysis, each region was segmented into 20 equal bins, with the value in each bin representing the mean methylation level calculated from all pertinent data within that specific interval

Differential DNA methylation patterns were prominent across various genomic regions in the TT, TE, and TP graftings (Table S8; Fig. 5a). In these graftings, the coding sequence (CDS) regions displayed the lowest levels of methylation, while the repeat regions exhibited the highest. Methylation in intron regions was relatively low, slightly overshadowed by that in promoter regions, with CpG islands exhibiting more substantial methylation. Notably, within these regions, CG methylation levels were consistent across all grafting types. However, CHG and CHH methylation levels were markedly higher in the TP grafting compared to the TT within the same regions.

Further exploration of the methylation distribution within functional gene regions, including the promoter, first exon, intron, last exon, and downstream regions, revealed additional complexities (Fig. 5b). Methylation levels were characteristically lower in coding regions, specifically the first and last exons, compared to non-coding regions. An intriguing dynamic was noted in the methylation patterns: CG methylation demonstrated a steady increase in the first exon, decreasing towards the last, with CHG and CHH patterns displaying the opposite trend. Besides, all three types of methylation showed a pronounced increase followed by a more gradual decrease in the promoter region, indicative of its sophisticated methylation pattern.

### Negative correlation between the methylation and transcription levels of the genes located in the differentially methylated regions (DMRs) of TP/TT grafting comparisons

**Fig. 6:**
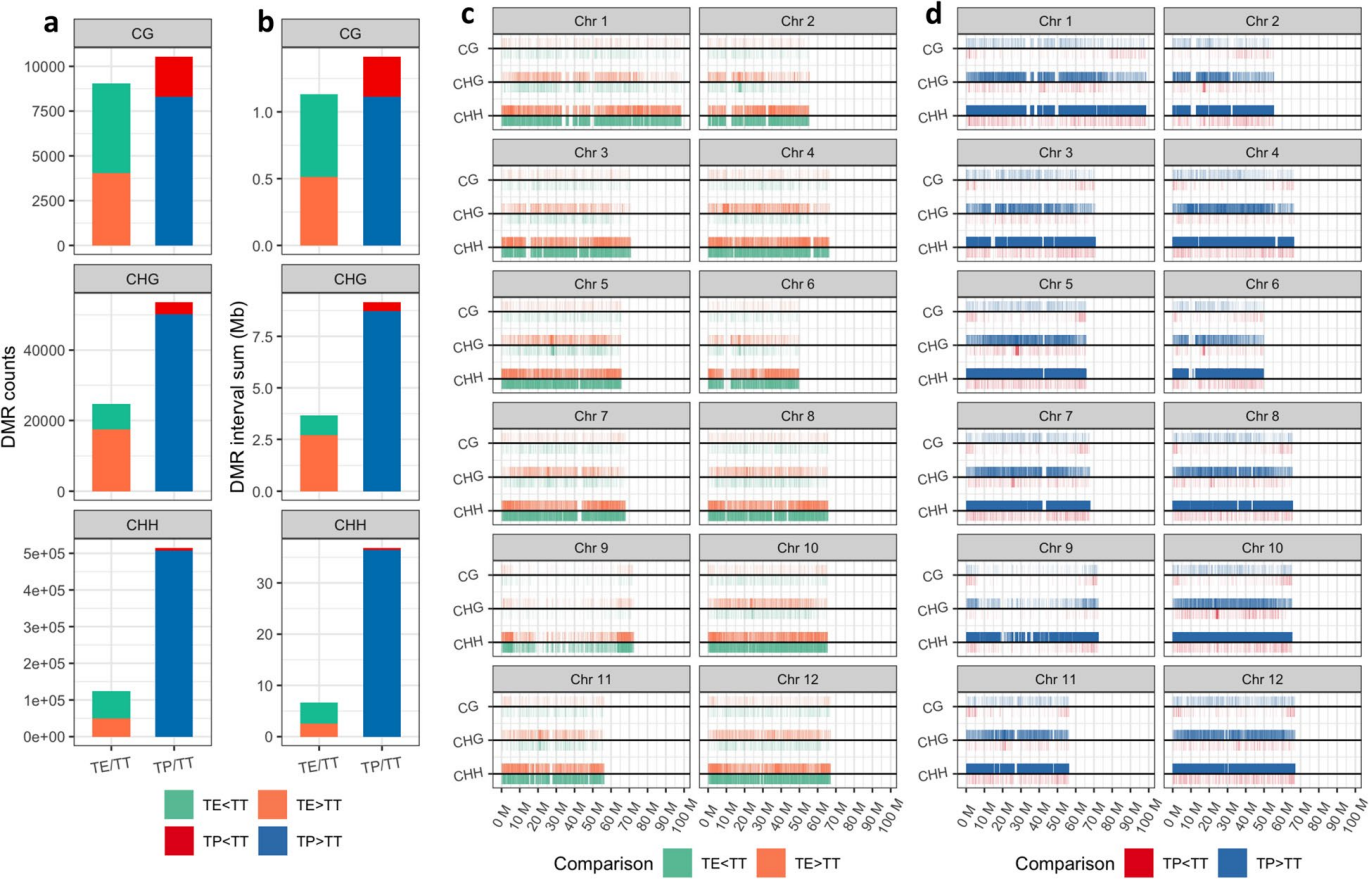
Analysis of genome-wide differentially methylated regions (DMRs) in the TE/TT and TP/TT grafting comparisons. **a** Number of hypermethylated and hypomethylated DMRs detected in the TE/TT and TP/TT grafting comparisons. **b** Total genomic length covered by hypermethylated and hypomethylated DMRs in the TE/TT and TP/TT grafting comparisons. **c** Chromosomal distribution of DMRs in the TE/TT grafting comparison. **d** Chromosomal distribution of DMRs in the TP/TT grafting comparison

An analysis of genome-wide DMRs between interspecific grafting and self-grafting (Table S9; Fig. 6a–b) revealed 70,446 hypermethylated and 87,358 hypomethylated DMRs in the TE/TT grafting comparison, covering areas of 5.66 Mb and 5.83 Mb, respectively. In addition, the TP/TT grafting comparison uncovered 566,784 hypermethylated and 11,668 hypomethylated DMRs, spanning 46.24 Mb and 1.13 Mb, respectively. This indicates a higher prevalence of genomic intervals with elevated CHG and CHH methylation levels in the TP grafting compared to that of the TT grafting (Fig. 6c–d).

Furthermore, an integrated analysis was carried out on the methylation and transcriptional levels of genes within the DMRs (Fig. S4). The results showed that no significant correlation between DNA methylation and transcriptional levels in the TE/TT grafting comparison. In contrast, a significant negative correlation was identified in the TP/TT grafting comparison. Specifically, genes within the TP/TT DMRs demonstrated negative correlations between their CG, CHG, and CHH methylation levels and transcriptional activity, with correlation coefficients of -0.193, -0.057, and -0.031, respectively.

### Modification in DNA methylation levels within the TP grafting play a crucial role in the activation of defense-related genes and the suppression of growth-related genes

Genes identified in the DMRs during a comparative analysis of the TP and TT graftings, exhibiting high methylation levels and low transcription levels in the TP grafting, were found to be enriched in growth-related pathways such as "RNA polymerase activity", "glycolipid metabolic process", "starch and sucrose metabolism", "carbon metabolism", and "fatty acid metabolism" (Fig. S5a). Conversely, genes exhibiting high methylation levels and low transcription levels in the TT grafting were found to be enriched in defense-related pathways such as "sesquiterpenoid metabolic process", "anion transmembrane transport", "carbohydrate derivative binding", "benzoxazinoid biosynthesis", and "glycosaminoglycan degradation" pathways (Fig. S5b).

Furthermore, the detailed methylation information of some growth and defense-related genes was examined. It was found that genes involved in DNA replication and transcription, such as DNA primase, DNA ligase, and RNA polymerase, exhibited multiple hypermethylated regions in the TP grafting compared to the TT grafting (Fig. S6a). Additionally, some kinase genes associated with the defense response displayed multiple hypomethylated regions in the TP grafting relative to the TT grafting (Fig. S6b). These findings suggest a crucial role for DNA methylation in the regulation of reallocating resources from growth to defense in the TP grafting.

## The rootstock-derived transcripts were identified in the scions of interspecific graftings

In the study, RNA-Seq data from the TE and TP graftings were mapped to the eggplant and pepper genomes, respectively. The analysis identified 649 and 337 rootstock-specific mobile transcripts in the TE and TP graftings, respectively, after scion reads were eliminated (Table S10). Of these transcripts, 58 in TE and 33 in TP were associated with defense responses, representing 8.93% and 9.79% of the total mobile transcripts, respectively (Fig. S7). Notably, within the defense-related transcripts transported in the TP grafting, 18 encoded serine/threonine protein kinases (Table 1). These findings suggest a potential underlying mechanism for the enhanced defense response observed in interspecific grafting compared to self-grafting.

**Table 1:** Transferred serine/threonine kinase transcripts from rootstock to tomato scion in the TP grafting

Gene ID	Description	FPKM	Gene ID	Description	FPKM
LOC107864188	serine/threonine-protein kinase	170.49	LOC107856315	calcium-dependent protein kinase 2	129.12
LOC107860687	serine/threonine-protein kinase STY13	111.54	LOC107878302	serine/threonine-protein kinase	72.01
LOC107850612	mitogen-activated protein kinase 9	58.30	LOC107878166	lysM domain receptor-like kinase 3	34.63
LOC107875929	serine/threonine-protein kinase	24.33	LOC107850317	cyclin-dependent kinase C-1	23.10
LOC107845711	receptor-like cytoplasmic kinase 2	18.38	LOC107861624	glycopeptide receptor 1	20.00
LOC107866776	serine/threonine-protein kinase IRE1a	16.18	LOC107850439	serine/threonine-protein kinase EDR1	8.56
LOC107861329	receptor-like protein kinase PERK1	3.24	LOC107855397	serine/threonine-protein kinase SIS8	2.67
LOC107845413	kinase THESEUS1	2.64	LOC107863419	serine/threonine protein kinase IRE	1.38
LOC107838744	phytosulfokine receptor 1	1.42	LOC107873067	probable receptor-like protein kinase	0.87

The FPKM values represent the mean FPKM quantification of two samples in the TP grafting

## Discussion

### Interspecific grafting induces enhanced scion resistance through wounding, endogenous immune pathways, and DNA methylation

Previous studies have demonstrated that grafting can effectively improve the environmental adaptability of grafted scions [19, 20]. In the present study, interspecific grafting within the *Solanaceae* family significantly enhances the resistance of tomato scions to gray leaf spot, offering a novel option for safeguarding tomato production beyond traditional breeding strategies. To reveal the dynamic changes of scions after grafting events, previous grafting-related research has mostly focused on the graft union between rootstock and scion, and the time points for study were kept short after grafting [13, 21]. In contrast, this study diverges by sampling tissues at extended intervals post-grafting, revealing substantial impacts on tomato scion growth and defense across diverse graft combinations.

In general, a wounding response is observable at the graft union between the rootstock and scion post-grafting, characterized by a series of biological reactions such as the activation of JA synthesis and increased ROS levels [22, 23]. In this study, wounding response related genes in the TT, TE, and TP grafting combinations exhibited a gradual upregulation (Fig. 3c), suggesting a progressively enhanced wounding response. Besides, external stressors like herbivore and insect activity, pathogen invasion, and plant parasitism have been known to similarly trigger wounding responses in plants, initiating akin defense mechanisms [24–26]. These insights are critical as they not only corroborate previous observations but also elucidate the potential mechanisms underlying the enhanced disease resistance observed in tomato scions following interspecific grafting.

JA is shown to play a pivotal role in plant responses to wounding and resistance. It responds to wound by activating the *ERF109* gene, which subsequently upregulates the *ASA1* gene and induces auxin production [27]. In wheat, the exogenous application of methyl jasmonate (MeJA) not only significantly enhances the activities of antioxidative enzymes, but also contributes to higher antioxidant enzyme activities and elevated levels of H_2_O_2_, resulting in a marked increase in wheat's resistance against the pathogen *Fusarium culmorum* [28]. Similarly, the application of JA and MeJA in tomatoes elevates the levels of defense enzymes such as polyphenol oxidase (PPO), effectively interfering with herbivore feeding [29]. These findings across different species underscore the versatile role of JA in plant defense mechanisms, demonstrating its importance across a diverse range of plant responses.

A critical aspect of this research involves the identification of key enzymes in the JA synthesis pathway. Studies have revealed that wounding or pathogen attack increases the activity of lipoxygenase enzyme, which initiates the jasmonic acid synthesis pathway, and OPDA reductase, responsible for reducing OPDA to OPC-8 [30, 31]. These enzymes showed significant transcriptomic differences in the TT/TE or TT/TP comparisons. However, the most related genes did not meet the conventional |log_2_ (fold change)| > 2 threshold for DEGs. Consequently, a more inclusive |log_2_ (fold change)| > 0.8 was adopted as the screening criterion for DEGs related to the JA synthesis pathway and yielded enriched outcomes. Although the selection criteria for these DEGs were broadened, the findings offer significant contributions to the understanding of JA-related processes in TE and TP graftings. Furthermore, our study revealed that wound-induced proteinase inhibitors, which are crucial for JA signaling [30], remained elevated in the TP grafting combinations. This sustained response in TP grafting highlights the integral role of JA in enhancing wound responses and disease resistance, particularly in interspecific graftings. These results are in alignment with previous studies, which have indicated the induction of these transcripts in the early stages of tomato wounding [30].

In response to bacterial and fungal invasions, plants utilize membrane-surface receptors to detect pathogen molecular patterns, inducing the PTI process, and subsequently leading to the upregulation of disease resistance genes [32]. This study revealed a significant upregulation of genes associated with kinase cascades in the PTI pathway in the TP grafting. However, these genes were not highly expressed in the TE grafting, indicating a potential variance in the defense responses activated between these two interspecific graftings. Furthermore, effector proteins secreted by invading pathogens can initiate a more potent ETI plant immune response within plant cells [33]. Notably, there were high expression of *CNL* and *TNL* transcripts, crucial signaling molecules in the ETI pathway, in both TE and TP graftings [32]. Regarding the downstream components of the *TNL* signaling pathway, while the EDS1-PAD4-ADR1 signaling node was highly expressed in both graftings, the EDS1-SAG101-NRG1 signaling node did not show similar expression patterns. It's also important to highlight that genes related to the *NPR1* immune pathway were not upregulated in either TE or TP graftings, suggesting that interspecific grafting may induce only partial responses in certain branches of the ETI pathway, a finding that prompts further investigation into the complex interactions within plant immune responses.

The interspecific grafting events in this study also induced high levels of ROS, in the TE and TP graftings compared to the TT grafting. ROS production is also regulated by *BIK1* and *PBL1*, which are associated with PTI and ETI immunity pathways [34]. Moderate levels of ROS are required for plant growth and immunity, while high levels of ROS can severely inhibit growth and even cause cell death in plants [35]. Besides, the movement of RNA molecules from rootstock to scion has been identified as a crucial factor in reshaping scion phenotypes [36, 37]. Notably, this study found that some transcripts, including serine/threonine kinases, were expressed in the scions from the rootstock in the TP grafting (Table 1), suggesting these mobile RNA transcripts traverse the rootstock-scion interface and exert ongoing influences on the scion's phenotypes.

Although grafting is an asexual reproduction technique, the expression of scion genes can be influenced by epigenetic regulation [17]. In this study, the TP grafting exhibited a high proportion of hypermethylated regions compared to the TT grafting, particularly in CHH methylation (Fig. 6). Unique to plants, CHH methylation plays a vital role in regulating growth, development, and defense responses [38, 39]. De novo methylation is achieved through the RNA-directed DNA methylation (RdDM) pathway, with the methylation transferase *DRM2* significantly catalyzing the methylation of cytosine at the 5th carbon [40], this process was notably active in the TP grafting (Fig. 3c). Furthermore, maintaining methylation levels is crucial for genetic stability [41], yet genes associated with methylation maintenance, such as *MET1*, *CMT2*, and *CMT3*, weren't expressed at high levels in the TP grafting, indicating a reliance on de novo methylation. Previous studies has underscored the role of RdDM methylation in plant antiviral defenses [42]. In Arabidopsis, the methyl-related mutants like *nrpd2* and *drm2*, showed an increase in susceptibility to pathogen infection [43], suggesting that DNA methylation is a pivotal strategy for plants in responding to biotic stress and serves as a key epigenetic factor differentiating the defense responses between the TE and TP graftings.

In general, gene regions exhibit a negative correlation between methylation levels and transcription levels [44]. Notably, in the TP grafting combination, several defense-related genes, including disease resistance kinases, showed low methylation levels alongside high transcription levels. This indicates that reduced methylation might play a role in the transcriptional upregulation of these key defense genes, as depicted in Fig. S6. In contrast, the comparison between the TE and TT groups did not reveal a significant correlation between overall gene transcription and methylation levels, as evident in Fig. S4. This absence of correlation might be due to the methylation levels in the TE group not reaching the same intensity as in the TP group, thus not eliciting a comparable change in transcription levels. This hypothesis is further corroborated by single-gene analyses, which demonstrate a significant negative correlation between transcription and methylation levels predominantly in the TP group, as detailed in Fig. S6.

Furthermore, the transcriptome analysis results of this study reveal a gradual increase in the expression of transcripts associated with water dehydration, wounding responses, and methylation in the TT, TE, and TP graftings. This trend indicates a significant correlation in the expression of genes involved in dehydration, wound response, and methylation processes. Specifically, in interspecific graftings, especially those involving incompatible combinations, issues with the vascular bundle connections between the rootstock and scion have been noted [45]. These vascular bundle connection issues may lead to a prolonged wound response in the scion, potentially impeding water transport and thus limiting its survival resources especially in the TP graftings. Consequently, the rootstock may adapt by reallocating resources towards a defensive state to counteract the stress, which includes enhanced responses to dehydration and increased methylation levels. Analogous studies in rubber trees have demonstrated that wound responses can trigger dehydration and JA synthesis, underscoring the generalizability of these findings [46].

### Interspecific grafting improves the fruit quality though it limits the growth resources

Grafting, an agricultural technique, utilizes the advantages of rootstock to alter scion phenotypes, contributing significantly to the enhancement of fruit quality and scion performance [47, 48]. This study discovered that interspecific grafting significantly boosted the levels of soluble solids and vitamin C in tomato fruits, surpassing the results of self-grafting. These observations are consistent with prior research, especially when wild tomatoes were used as the rootstock [47], highlighting the potential of improved fruit nutritional quality in interspecific graftings. Nevertheless, the TE and TP graftings exhibited inhibition in growth and fruit yield, indicative of different degrees of graft incompatibility. Graft incompatibility is common in grafts involving rootstock and scion with considerable genetic disparity [49]. This is evident in the TP grafting, given the genetic distance between the pepper rootstock and tomato scion; while eggplant (*Solanum melongena* L.) and tomato (*Solanum lycopersicum* L.) both belong to the same *Solanum* genus, pepper (*Capsicum annuum* L.) belongs to the *Capsicum* genus [50]. Incompatibility often leads to discontinuous vascular bundles at the graft union, disrupting water and nutrient transport [45], likely contributing to the inhibited growth in the TE and TP graftings in this study.

Additionally, it was observed that processes crucial to the cell cycle, including DNA replication, cytokinesis, and cell wall synthesis, were notably inhibited in the TP grafting. This inhibition encompasses the NACK-PQR pathway, crucial for mitotic initiation, triggered by NACK and leading to downstream cytokinesis through a mitogen-activated protein kinase cascade [51]. The transcriptional levels of *NACK* genes were significantly suppressed in the TP grafting (Fig. 4). Furthermore, kinesin microtubule proteins, which are vital for cell division [52], exhibited low transcriptional levels in the TP grafting (Fig. S2). A gradient decrease was also evident in the transcriptional levels of ribosomal and chlorophyll proteins related to energy metabolism and photosynthesis across the TT, TE, and TP grafting combinations. This pattern suggests constrained nutrient accumulation in the scions of TE and TP graftings, aligning with the observed reductions in leaf and fruit sizes.

### The allocation of resources for scion growth and defense determines the grafting states

The balance between allocating resources for scion growth and defense is pivotal in determining grafting outcomes. This study noted resistance to tomato gray leaf spot in both TE and TP graftings, despite the observed growth inhibition in the TP grafting. This phenomenon seems to reflect the growth-defense tradeoff, a well-documented antagonistic relationship in plant biology where resources are meticulously balanced between growth and defense mechanisms, particularly under conditions of limited resources or stress [53, 54]. In the TP grafting, the tomato scions, faced with survival challenges due to graft incompatibility, appeared to prioritize defense over growth. This shift was evidenced by sustained wounding responses, elevated DNA methylation levels, and robust endogenous plant immunity, while processes fundamental for growth—such as the cell cycle, DNA replication, cytokinesis, and cell wall synthesis—were markedly suppressed. In contrast, the TE grafting demonstrated a more harmonious balance of resources, accommodating both standard scion growth and disease resistance. These observations underscore the intricate biological calculus plants must navigate in allocating resources, a consideration that holds significant implications for agricultural strategies and understanding plant resilience.

**Fig. 7:**
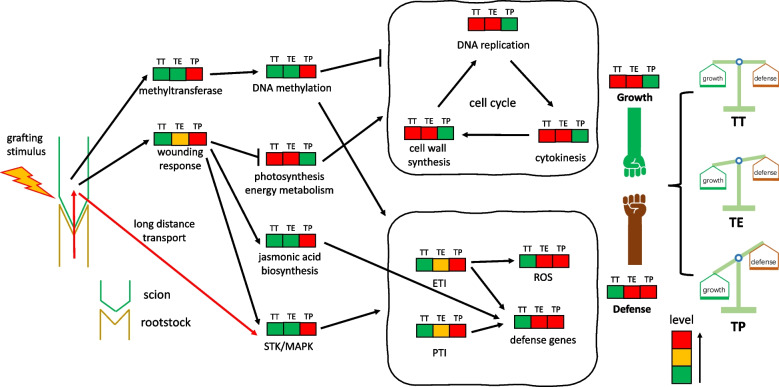
Applying the growth-defense trade-off theory to tomato grafting with different rootstocks. The red arrow indicates rootstock-specific mobile transcripts in interspecific graftings. The green, orange, and red color blocks respectively represent physiological activity processes at low, medium, and high levels. Overall, the grafting combinations with different rootstocks correspond to varying degrees of trade-off between growth and defense resources

Previous studies on the 'growth-defense trade-off' have primarily focused on plant responses to environmental abiotic stresses [55] and biotic stresses from insect herbivory or pathogen infection [56]. This study furthers these investigations by demonstrating that interspecific grafting can also lead to limited survival resources in the scion, influencing the balance of growth and defense. The compatibility between rootstock and scion dictates the allocation of survival resources, skewing the balance from growth to defense. In this context, the grafting stress experienced in the TE and TP graftings appears to activate the scion's ETI and PTI pathways, crucial for biotic stress resistance, as evidenced in *chrysanthemum* when *Artemisia scoparia* W. was used as a rootstock for aphid defense [6]. However, excessive grafting stress also resulted in graft incompatibility (Fig. 7), limiting growth resources in the scion and negatively impacting tomato production. Thus, it's imperative to maintain grafting stress within acceptable limits to optimize outcomes in tomato grafting. This study contributes to the theoretical framework of the growth-defense trade-off for evaluating tomato grafting rootstocks.

In previous studies, tomato grafting experiments primarily focused on mitigating soil-borne diseases, such as *Sclerotium rolfsii* [57]. In this study, it has been discovered that TE and TP grafting combinations can induce a defense response against gray leaf spot, a non-soil-borne disease, potentially offering more promising grafting strategies for protected cultivation of tomatoes. Moreover, the choice of rootstock is crucial for the performance of the scion. Despite the apparent graft incompatibility in the TP grafting observed in this experiment, it provided a wealth of phenotypic and multi-omics data for exploring the growth-defense trade-off theory. Therefore, we retained the results of this grafting for detailed examination. Future studies will consider a broader range of rootstock options. For example, exploring more grafting combinations using wild tomato species *Solanum pimpinellifolium* as rootstocks, which aims to find a better balance between growth and defense in scions and through extensive field grafting trials, to explore more effective rootstock-scion combinations. These efforts are directed towards providing viable grafting solutions for the sustainable production of tomatoes in protected cultivation.

## Conclusions

This study demonstrates that grafting tomato scions onto eggplant and pepper rootstocks can significantly boost resistance to gray leaf spot, albeit with a reduction in fruit yield compared to self-grafting. Through comprehensive transcriptome and methylation analysis, it has been discovered that interspecific grafting initiates sustained wound responses and activates innate immune pathways. This process is accompanied by a more pronounced dehydration response and high methylation level, particularly evident in TP grafting. However, it also inhibits cell cycle-related pathways, including DNA replication, cytokinesis, and cell wall synthesis, especially in the TP (tomato/pepper) grafting. Notably, TP grafting presented a substantial increase in genome-wide hypermethylated DMRs relative to TT (tomato/tomato) self-grafting, implicating genes associated with DNA replication and other cell cycle processes within these DMRs. Additionally, disease-resistant kinases in TP grafting exhibited a tendency towards lower methylation and higher expression. These findings suggest that interspecific grafting dynamically modulates the balance between growth and defense resources in the scion by altering gene methylation levels, thereby enhancing disease resistance at the cost of growth inhibition. Furthermore, this study broadens the applicability of the growth-defense trade-off theory to grafting experiments.

## Materials and Methods

### Tomato grafting using tomatoes, eggplants, and peppers as rootstocks

A susceptible tomato variety to gray leaf spot "Jinfen 207" was used as scions, and three common *Solanaceous* breeding lines from China, including tomato line "Jinfen 207", eggplant line "Kuaiyuan Qie", and pepper line "Golden A", were used as rootstocks. The tomato self-grafting was performed from different plants of the same variety. Tomato, eggplant, and pepper cultivars were individually grown in 50-cell trays (single cell size: 5.0 cm × 5.0 cm × 5.0 cm) under a photoperiod of 16 h light/8 h dark and a temperature of 25 °C/15 °C for 40, 60, and 60 days before grafting, respectively. Uniform seedlings were selected for grafting. Grafting operation was performed using tomato as the scion and tomato, eggplant, and pepper as rootstocks. All scions and rootstocks were cut at a 45-degree angle, and the cut surfaces were secured together using plastic clips to create three grafting combinations, including one self-grafting (TT), and two interspecific graftings (TE and TP).

### Gray leaf spot resistance and growth characteristics evaluation of grafted tomato scions

At 21 days of post-grafting, a homogeneous suspension of *Stemphylium* spores was sprayed to the abaxial (underside) surfaces of the grafted tomato scion leaves using a handheld sprayer. Each set of ten plants received a 10 ml spore suspension, ensuring even coverage of the leaf surfaces to induce gray leaf spot. This application was repeated daily for three consecutive days. Fourteen days following the final spray, the disease incidence in the grafted tomato scions was observed and recorded. *Stemphylium* strains were purified and inoculated on PDA medium for 10 days, then transferred to the sporulation medium for 8 days. *Stemphylium* spores were centrifuged (6000 rpm, 10 min) to remove the supernatant and were diluted using sterile water to 10^5^ spores per milliliter. After 30 days of grafting, the first fully expanded tomato leaves, starting from the apical meristem were collected as samples, and the accumulation of O_2_^−^ and H_2_O_2_ in the sample leaves was measured using the NBT and DAB staining methods described by [58].

TT, TE and TP grafting combinations were transplanted into the greenhouse after 14 days of grafting at the Tianjin Academy of Agricultural Sciences (Tianjin, China). Each grafting was divided into two replicates, with ten plants per replicate. The yield of tomatoes was calculated by harvesting mature fruits within 3 clusters. Five mature tomato fruits were randomly selected from each grafting combination. A caliper was used to measure the diameters of tomato fruits. The weight of the tomato fruits was measured using a digital scale. The soluble solid content in mature tomato fruits from the second scion of each grafting combination was determined using a portable fruit refractometer (ATAGO Co., Ltd., Japan). The refractometer was first calibrated with distilled water. Subsequently, 0.3 ml of juice extracted from the grafted tomato fruits was applied to the refractometer's prism for analysis. Three fruits from each grafting combination were tested, each serving as a separate replicate. The vitamin C content in the grafted tomato fruits was quantified using a Vitamin C measurement kit (LEAGENE company, China). For each grafting combination, three replicates were prepared by grinding 30 g of tomato fruit and diluting with 1 × homogenization buffer to a final volume of 50 ml. After thorough mixing, the homogenate was centrifuged at 10,000 g for 5 min, and 0.5 ml of the supernatant was collected for measurement. In the vitamin C assay, a standard curve correlating Vitamin C concentration to absorbance was established using the data from the blank and standard tubes, and the vitamin C content in the samples was calculated from the absorbance difference between Test Tubes I and II. Detailed preparation instructions for the blank, standard, and test tubes (Test Tubes I and II) are provided in the accompanying manual.

### RNA sequencing and data analysis

In this study, gene transcription levels in tomato scions were examined following grafting. Specifically, the first fully expanded leaf located below the growth point was sampled from each of the TT, TE, and TP grafting scions. These samples were collected 30 days post-grafting, each from two independent biological replicates. The total RNA of samples was extracted using the RNeasy Kit (Qiagen, China), followed by mRNA enrichment using oligo (dT). Furthermore, the enriched mRNA was subjected to fragmentation, reverse transcription, and PCR amplification for RNA-seq libraries preparation. All RNA-seq libraries in this study were sequenced on the BGISEQ-500 platform (Beijing Genomics Institute, BGI, China), with 150 bp paired-end reads. Raw sequencing reads were filtered using SOAPnuke software (https://github.com/BGI-flexlab/SOAPnuke) to remove low-quality reads with default parameters.

The filtered clean reads were aligned to the tomato genome (*Solanum lycopersicum* L., version: SL2.50) using HISAT2 software as described by [59]. Subsequently, the reads were quantified using StringTie software [60], and DEGs between grafting combinations were identified using DEseq2 package [61], according to the criteria of |log_2_ (fold change)|> 2 and q value < 0.001. DEGs that were significantly enriched in GO terms and KEGG pathways [62] were analyzed using the clusterProfiler package in R software, as outlined by [63].

### Detection of transcripts mobilized from rootstock to scion

The transcriptome sequencing reads of tomato scions used eggplant and pepper as rootstocks (TE and TP graftings) were aligned to the eggplant (*Solanum melongena* L., version: SMR_2.5.1, http://eggplant.kazusa.or.jp) and pepper (*Capsicum annuum* L., version: UCD10Xv1.1,https://ftp.ncbi.nlm.nih.gov/genomes/refseq/plant/Capsicum_annuum/latest_assembly_versions/GCF_002878395.1_UCD10Xv1.1/) genomes using HISAT2 software, respectively. The mapped reads were quantified using StringTie software. The interfering transcripts from scion was eliminated by constructing a BLAST nucleotide library using tomato mRNA sequences. The eggplant and pepper mRNA sequences were used as query sequences for local BLAST searches. Transcripts with high similarity between the rootstock and scion were filtered out and excluded based on the following criteria: an E-value lower 1e-50 or a score exceeding 200 for the TE grafting, alongside an E-value below 1e-50 or a score greater than 300 for the TP grafting. The remained transcripts were noted as the rootstock-specific mobile transcripts.

### Validation of the gene expression level by qRT-PCR

The differential expression levels of a select group of representative genes identified through RNA-sequencing were further validated by qRT-PCR. The primer sequences of the selected genes are available in Table S11. The total RNA was reverse transcribed using M-MLV reverse transcriptase (TAKARA, Japan), and all qRT-PCR analysis were conducted on a Real-Time PCR fluorescence detection system (5698R-1244, BIO-RAD, America) in three replicates. The 2^−∆∆ct^ method was used to calculate gene expression fold changes among groups, with the housekeeping gene *Solanum lycopersicum* actin-7-like (LOC101262163) was utilized as an internal control [64].

### The WGBs-seq and DNA methylation analysis

The DNA methylation patterns and levels in restructuration of tomato grafting scions was investigated by the WGBs-seq, with two independent biological replicates in each group. Genomic DNA from the first fully expanded leaf of the TT, TE and TP scions was extracted using the CTAB method [65] and subsequently fragmented to approximately 250 bp using a bioruptor (Diagenode, Belgium). After completing the end repair, A-tailing, and adapter ligation processes, the DNA fragments were subjected to bisulfite treatment and subsequently purified using the Methylation-Gold kit (ZYMO, America). The DNA fragments were then amplified by PCR, and the reads ranging from 320 to 420 bp were used for library preparation. All WGBs libraries were sequenced on the Hi-Seq 2500 (Illumina, America) platform using the 150 bp paired-end method by the BGI Company (Shenzhen, China).

The WGBS-seq raw reads were filtered using SOAPnuke software by removing low quality reads. The filtered clean reads were stored in the FASTQ file. The clean reads were converted to their bisulfite-converted versions (C- > T, G- > A) and were mapped to the tomato reference genome (*Solanum lycopersicum* L., version: 2.50) using BSMAP software [66]. The bisulfite conversion rate (*r*) and DNA methylation level were calculated after eliminating duplicate reads as per [67].

The cytosine DNA methylation (5-methylcytosine, 5mC) was classified into CG, CHG, and CHH contexts, and the methylation levels of these three contexts on each chromosome were calculated using the R software (https://www.r-project.org). Chromosome-level methylation curves were calculated using the sliding-window approach with a 500 kb bin width and a 1 kb step. Furthermore, the methylation levels of CG, CHG, and CHH in various gene regions were calculated, including promoters (0–1.5 kb upstream of Open Reading Frames), first exons, introns, last exons, and downstream regions (0–0.5 kb downstream of Open Reading Frames). Each region was divided into 20 bins, and the average methylation level within each bin was calculated by the mean methylation of all genes in the corresponding bins.

### DMRs detection and the enrichment of the genes located within DMRs

The genome region containing at least 5 CG (CHG or CHH), and the significant methylation differences (twofold difference, and Fisher's test *p*-value < 0.05) between grafting combinations were defined as DMRs. Two contiguous DMRs were merged into one DMR if these two DMRs both had significant differences between the grafting combinations. The correlation between the methylation levels and transcription levels of genes within all the DMR regions of grafting combinations was performed. Additionally, the enrichment analyses of GO terms and KEGG pathways were also conducted for the genes located within the DMRs.

### Supplementary Information

**Additional file 1.** Tables S1-S11

**Additional file 2.** Figures S1-S7

## Data Availability

The clean reads of transcriptome and DNA methylome datasets generated and analyzed during the current study are available in the NCBI database (https://www.ncbi.nlm.nih.gov), and the BioProject IDs are PRJNA1020285 and PRJNA1019841 respectively. All other data generated or analyzed during this study are included in this published article and its supplementary information files.

## Abbreviations

TT: The grafting of tomato/tomato
TE: The grafting of tomato/eggplant
TP: The grafting of tomato/pepper
PTI: Pattern-triggered immunity
ETI: Effector-triggered immunity
ROS: Reactive oxygen species
NBT: Nitroblue tetrazolium
DAB: 3,3'-Diaminobenzidine
DEGs: Differentially expressed genes
GO: Gene ontology
KEGG: Kyoto Encyclopedia of Genes and Genomes
JA: Jasmonic acid
MeJA: Methyl jasmonate
qRT-PCR: Quantitative reverse-transcription PCR
WGBs-seq: Whole-genome bisulfite sequencing
CDS: Coding sequence
DMRs: Differentially methylated regions
