# Supplementary material for: Integrated transcriptome and DNA methylome analysis reveal the biological base of increased resistance to gray leaf spot and growth inhibition in interspecific grafted tomato scions
Source: BMC Plant Biol. 2024 Feb 21;24:130. doi: 10.1186/s12870-024-04764-8 (PMC10880203; doi:10.1186/s12870-024-04764-8)
Supplement: Supplementary file 2 — Additional file 2. Figures S1-S7 [file 12870_2024_4764_MOESM2_ESM.docx]

Additional file 2

Fig. S1 GO and KEGG pathway enrichment analysis of DEGs in the TE/TT and TP/TT grafting comparisons. This enrichment analysis shows the functional biological processes and pathways impacted by interspecific grafting compared to self-grafting. (a) Enrichment results for upregulated DEGs in the TE /TT grafting comparison. (b) Enrichment results for upregulated DEGs in the TP/TT grafting comparison. (c) Enrichment results for downregulated DEGs in the TE/TT grafting comparison. (d) Enrichment results for downregulated DEGs in the TP/TT grafting comparison. Enrichment analysis of KEGG pathways in this figure is based on the Kyoto Encyclopedia of Genes and Genomes database (https://www.kegg.jp/kegg/) [55].

Fig. S2 DEGs associated with ribosomal function, kinesin activity, and chlorophyll proteins in the TT, TE, and TP grafting combinations. This heatmap highlights the variability in gene expression levels related to crucial biological processes and photosynthesis efficiency in different grafting scenarios. The analysis focuses on DEGs critical for ribosomal function, kinesin activity, and chlorophyll protein production, offering insights into the physiological adaptations of the grafted plants. In the heatmap, colors correspond to normalized Z-score values, indicating the expression level of each gene relative to the mean expression across the samples: red denotes higher expression, and green represents lower expression. The columns of the heatmap, ordered from left to right, correspond to the TT, TE, and TP graftings, respectively.

Fig. S3 DNA methylation levels within each chromosome in the TT, TE, and TP graftings. (a) The bar plot of the average methylation levels of CG, CHG, and CHH across each chromosome, including chloroplasts; (b) The curves of the methylation patterns for CG, CHG, and CHH across each chromosome, analyzed using the sliding window method with a 500 kb window size and a 1 kb step size to highlight regional variations in methylation.

Fig. S4 Correlation between transcription and DNA methylation differences of the genes located in DMRs for the TE/TT and TP/TT grafting comparisons. The x-axis shows the logarithmic differences in gene transcription levels between groups, and the y-axis shows the corresponding differences in DNA methylation levels. Pearson correlation coefficients are indicated (***, **, and * representing *p*-values < 0.001, < 0.01, and < 0.05, respectively). Red dots signify positive correlations, and blue dots signify negative correlations between gene expression and DNA methylation differences. The black line depicts the linear regression between these logarithmic differences.

Fig. S5 Enrichment analysis of genes demonstrating inverse DNA methylation and transcription in comparison in the TP versus TT graftings. (a) Shows the enrichment analysis of the genes exhibiting higher transcription and lower DNA methylation in TP compared to TT graftings. (b) illustrates the enrichment analysis of the genes exhibiting lower transcription and higher DNA methylation in TP compared to TT graftings. The enrichment analysis incorporates three standard gene ontology domains — BP (Biological Process), CC (Cellular Component), and MF (Molecular Function) — along with Kyoto Encyclopedia of Genes and Genomes (KEGG) pathways, a comprehensive database resource essential for interpreting the complex functions and utilities of biological systems. Enrichment analysis of KEGG pathways in this figure is based on the Kyoto Encyclopedia of Genes and Genomes database (https://www.kegg.jp/kegg/) [55].

Fig. S6 Differential analysis of transcription and DNA methylation levels in selected genes among TT, TE, and TP graftings. This figure presents a comparative analysis of specific genes exhibiting differential transcription and DNA methylation levels among three grafting combinations. (a) Highlights genes in the TP grafting with downregulated transcription levels and increased DNA methylation levels compared to those in the TT and TE graftings; (b) focuses on genes in the TP grafting with downregulated transcription levels but decreased DNA methylation levels relative to TT and TE graftings. Functional element regions are divided into 20 bins, with each bin's value representing the mean of all pertinent data within that interval. In (a), arrows (indicated in red) pinpoint intervals experiencing an obvious rise in DNA methylation levels in the TP grafting, while in (b), arrows (indicated in orange) identify intervals with a notable reduction in DNA methylation levels in the TP grafting.

Fig. S7 Venn diagram of the resistance related transcripts that were transferred from rootstocks to tomato scions in the TE and TP graftings. (a) Transported genes related to resistance in the TE grafting, (b)Transported genes related to resistance in the TP grafting.
